# Evaluation of phenyl hydrazide-based compounds as myeloperoxidase inhibitors

**DOI:** 10.1007/s00210-025-04926-x

**Published:** 2026-01-29

**Authors:** Laryssa C. C. L. Salema, André B. Farias, Tiago R. Navarro, Patrick P. Pimentel, Thuany B. S. Aguiar, Nelilma C. Romeiro, Evanoel C. de Lima, Juliana M. Raimundo, Leandro L. da Silva

**Affiliations:** 1 Grupo de Pesquisa em Farmacologia de Produtos Bioativos (FarPBio), Instituto de Ciências Farmacêuticas, Centro Multidisciplinar UFRJ-Macaé, Universidade Federal do Rio de Janeiro, Av. Aluizio da Silva Gomes 50, Macaé, RJ 27930-560 Brazil; 2 Laboratório Integrado de Computação Científica (LICC), Instituto Multidisciplinar de Química, Centro Multidisciplinar UFRJ-Macaé, Universidade Federal do Rio de Janeiro, Macaé, RJ Brazil; 3 Laboratório Nacional de Computação Científica (LNCC), Petrópolis, RJ Brazil; 4 Laboratório de Catálise e Síntese de Substâncias Bioativas (LACASB), Instituto Multidisciplinar de Química, Centro Multidisciplinar UFRJ-Macaé, Universidade Federal do Rio de Janeiro, Macaé, RJ Brazil; 5 Grupo de Pesquisa em Farmacologia de Produtos Bioativos (FarPBio), Instituto de Ciências Médicas, Centro Multidisciplinar UFRJ-Macaé, Universidade Federal do Rio de Janeiro, Macaé, RJ Brazil

**Keywords:** Chronic diseases, Inflammation, Oxidative stress, Peroxidases, Hydrazide

## Abstract

Myeloperoxidase (MPO) has been associated with a broad range of chronic inflammatory diseases since activated MPO catalyzes the production of HOCl, triggering both oxidative stress and inflammation. Since benzohydrazides are known as MPO inhibitors, a series of six *N*'-phenylbenzohydrazides and one quinazolin-4(3*H*)-one analogue was evaluated through in vitro assays to measure inhibition of MPO, superoxide dismutase (SOD), and catalase (CAT) activities, as well as DPPH and superoxide-scavenging effects. Pharmacokinetic parameters of the compounds were evaluated in silico*,* and molecular docking studies were performed to predict their binding modes with the catalytic site of MPO. All phenylbenzohydrazides exhibited potent inhibitory effect on MPO activity, with four of them presenting IC_50_ values below 0.5 μM. Bromine substitution, as in **2b**, **2e**, and **2f**, enhanced the binding affinity to the active site of MPO, resulting in high inhibitory potency. In concentrations as high as 50 μM, compounds did not inhibit SOD activity but showed some inhibitory activity over CAT. Except for compound **2c**, the phenylbenzohydrazides can scavenge DPPH radical, while only **2e** can sequester superoxide anion. Quinazolin-4(3*H*)-one analogue **3**, obtained from phenylbenzohydrazide **2a** showed a more selective profile towards MPO compared to CAT but antioxidant activity was not observed. Phenylbenzohydrazide **2e** is a potent MPO inhibitor with antioxidant properties, demonstrated through DPPH and superoxide anion scavenging, with high predicted gastrointestinal absorption. The results obtained in this study with *N*’-phenylbenzohydrazides highlight the importance of structural modifications to improve the pharmacological profile of phenylhydrazides, contributing to the development of new anti-inflammatory drugs.

**Supplementary Information:**

The online version contains supplementary material available at 10.1007/s00210-025-04926-x.

## Introduction

Inflammation is a natural biological response of the body that occurs after cellular damage caused by microorganisms, physical agents, chemicals and/or immunological reactions. Normally, during acute inflammatory responses, cellular and molecular events efficiently minimise tissue injury or infection (Leuti et al. 2020).

On the other hand, chronic inflammation has been shown to play an important role in the development and progression of chronic diseases (Furman et al. 2019; Chen et al. 2018). In this case, the actors involved in immune vigilance and host defence mechanisms, such as the enzyme myeloperoxidase (MPO), contribute to tissue harm. Activated neutrophils can migrate to the inflammatory site even without the presence of pathogens, where MPO can be released into extracellular fluids (Herrero-Cervera et al. 2022).

Although MPO activation can result in the production of different oxidant products, hypochlorous acid (HOCl) is considered the most important because of the high concentration of Cl^−^ in human plasma. This potent oxidizing agent leads to oxidative stress and activation of metalloproteinases, resulting in tissue damage (Hawkins and Davies 2021). Several works have shown the involvement of MPO in the pathology of a variety of diseases associated with systemic chronic inflammation, such as cardiovascular, respiratory and neurodegenerative diseases, indicating the potential of this enzyme as a pharmacological target of new anti-inflammatory drugs (Herrero-Cervera et al. 2022; Davies and Hawkins 2020; van der Veen et al. 2009; Malle et al. 2007).

**Fig. 1:**
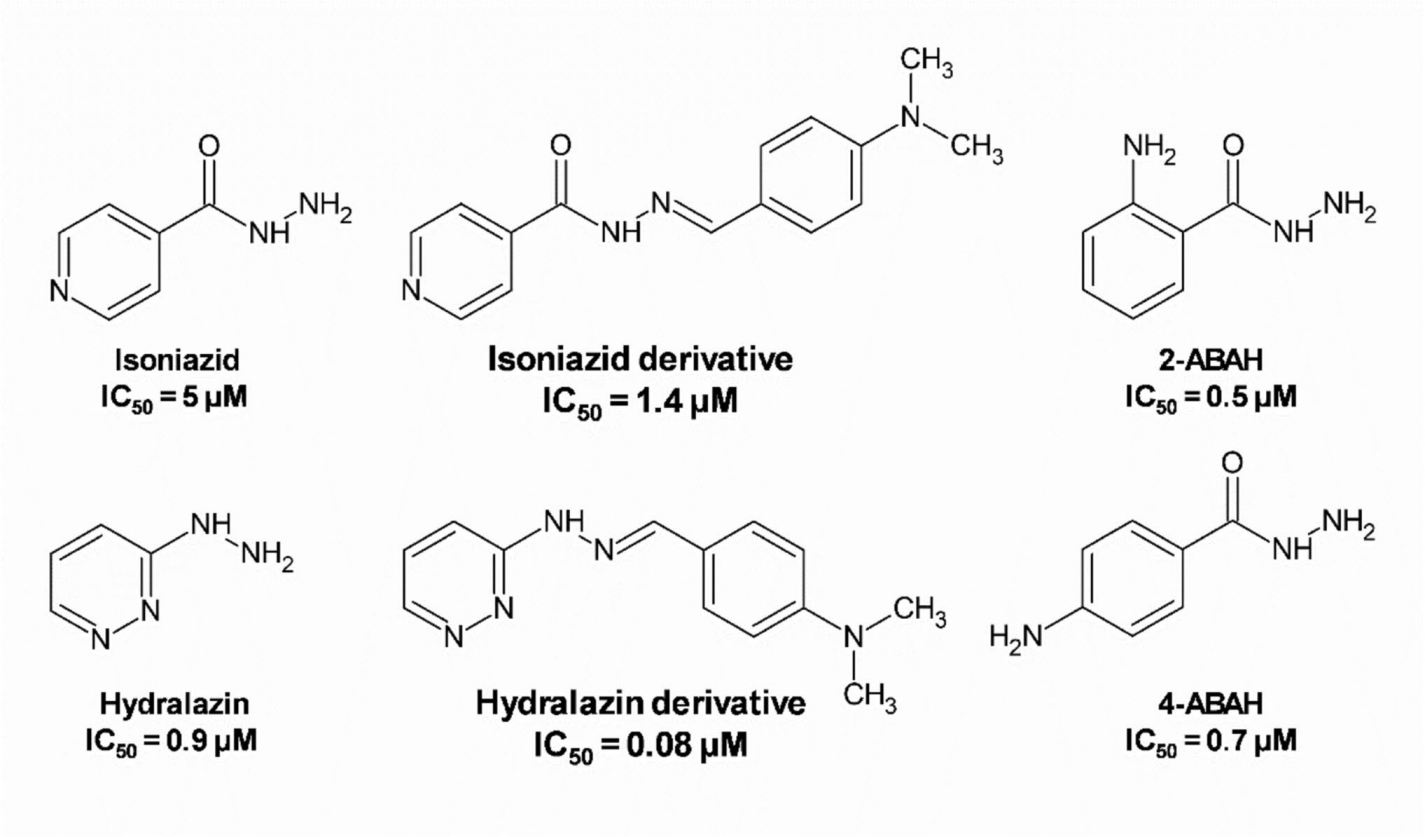
Chemical structures and potence of myeloperoxidase inhibitors

Hydrazides and hydrazines such as isoniazid (van Zyl et al. 1989), 4-Aminobenzoic acid hydrazide (4-ABAH) and 2-Aminobenzoic acid hydrazide (2-ABAH) (Kettle et al. 1995; Huang et al. 2014), and hydralazine (Soubhye et al. 2016) are known as MPO inhibitors. These compounds have been used to obtain hydrazones and hydrazide-hydrazones that also inhibit MPO activity, like isoniazid derivatives (Santos et al. 2020a, b) and hydralazine derivatives (Soubhye et al. 2016) (Fig. 1). 4-ABAH has been widely used to elucidate the role of MPO in different pathological conditions, including aneurysm (Shi et al. 2024), stroke (Forghani et al. 2015; Kim et al. 2016), non-alcoholic steatohepatitis (Pulli et al. 2015) and demyelinating diseases (Forghani et al. 2012).

Despite the structural resemblance of *N*’-phenylbenzohydrazides to known MPO inhibitors, the activity of this class of compounds has not been described yet. Therefore, in this work, we evaluated a series of *N*’-phenylbenzohydrazides and a quinazolin-4(3*H*)-one analogue as MPO inhibitors and antioxidant agents using in vitro and in silico assays. The synthesis of the compounds tested herein was previously described by co-author E.C. Lima. Specifically, hydrazides **2a–2d** were reported by Paiva et al. (2022), while compounds **2e**, **2f**, and **3** were described by do Carmo Maquiaveli et al. (2023).

## Materials and methods

### Synthesis of compounds

**Fig. 2:**
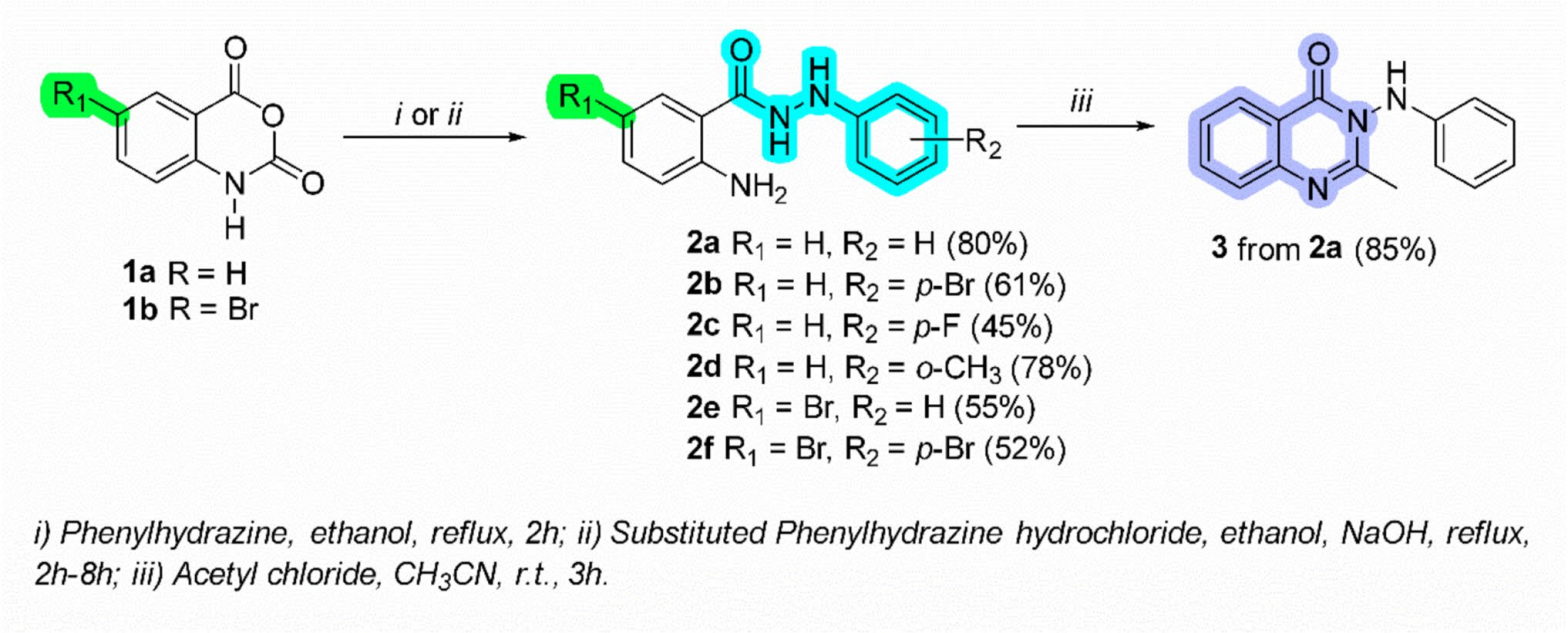
Scheme of reactions and structure of the studied compounds

*N*’-phenylbenzohydrazides 2-amino-*N*'-phenylbenzohydrazide (**2a**), 2-amino-*N*'-(4-bromophenyl)benzohydrazide (**2b**), 2-amino-*N*'-(4-fluorophenyl)benzohydrazide (**2c**), 2-amino-*N*'-(2-methylphenyl)benzohydrazide (**2d**), 2-amino-5-bromo-*N*'-phenylbenzohydrazide (**2e**), and 2-amino-5-bromo-*N*'-(4-bromophenyl)benzohydrazide (**2f**) were prepared using isatoic anhydride and respective phenyl hydrazines under reflux in ethanol for 2 h-5 h. Reaction to obtain 3-anilino-2-methylquinazolin-4(3*H*)-one (**3)** was carried out from phenyl hydrazide **2a** using acetyl chloride at room temperature as previously reported (Paiva et al. 2022; do Carmo Maquiaveli et al. 2023) (Fig. 2).

### In vitro* enzymatic and antioxidant assays*

#### MPO activity assay

This assay monitors 5-thio-2-nitrobenzoic acid (TNB) oxidation in an MPO/H_2_O_2_/Cl^−^/multi-substrate system. In 96-well plates, at room temperature, the MPO-rich homogenate, without (control) or with compounds (0.03—30 µM), was mixed with 140 mM NaCl, 50 μM NaSCN, 50 μM tyrosine, 200 μM urate, 1 mg/mL bovine serum albumin and 50 μM TNB in 20 mM phosphate buffer (pH 7.4). After 15 min, reactions were then initiated by adding 50 μM H_2_O_2_. Absorbance at 412 nm was monitored at 1-min intervals, for 10 min. The maximum reaction rate was calculated through 5 consecutive readings over 5 min (Forbes and Kettle 2018).

#### DPPH radical scavenging assay

In a 96-well plate, 200 µM DPPH and compounds (6.25—200 µM), solubilized in methanol, were kept under stirring at room temperature and protected from light for 30 min. Then, a spectrophotometer reading was taken at 517 nm, with the optical density obtained directly proportional to the amount of remaining DPPH. The control group consisted of DPPH and methanol (Sharma and Bhat 2009).

#### Superoxide scavenging assay

The superoxide-scavenging activity was evaluated according to a published procedure (Li 2012), with some modifications. In a 96-well plate, 0.5 mM pyrogallol was added to compounds (100 μM) in 10 mM phosphate buffer pH 7.4, with a final volume of 300 μL. After shaking, absorbance was measured at 355 nm every minute for 10 min at room temperature.

#### Superoxide dismutase (SOD) activity assay

Rat livers were homogenised with a Potter homogenizer in 0.1 M potassium-phosphate buffer, pH 7.4 (30 mL of buffer per 2.5 g of tissue). Successive centrifugations at 536 G, 7080 G and 15,000 G, for 10 min each, were performed to obtain the post-mitochondrial supernatant containing the copper, zinc SOD (Cu,ZnSOD). The supernatant was frozen until use. In a 96-well plate, compounds (50 µM) were incubated with 20 µL of the Cu,ZnSOD-rich supernatant in 0.2 M phosphate buffer (pH 8.0, with 2 mM EDTA) in a final volume of 295 µL. After 30 min of incubation, 5 µL of pyrogallol (60 mM) was added and its oxidation was monitored for 10 min at 1-min intervals at 420 nm (Campos-Shimada et al. 2020).

#### Catalase (CAT) activity assay

Bovine liver catalase (20 µg/mL) was incubated for 30 min with compounds (50 µM) in phosphate buffer solution (pH 7, 50 mM). Then, 1 mL of this mixture was added to the cuvette, where it was mixed with 1 mL of 0.072% H_2_O_2_ in phosphate buffer solution. H_2_O_2_ consumption by catalase was monitored in a spectrophotometer at 240 nm for 1 min with readings every 2 s (Krych and Gebicka 2013).

### In silico* studies*

#### Molecular docking analysis

First, the molecular structures of the ligands were constructed using Gaussian View v. 5.0.8 and energy was optimized using the PM3 method available in Gaussian03 (Frisch et al. 2004). The protein structure was obtained from the Protein Data Bank (Berman 2000) under the code 4C1M (Forbes et al. 2013). The protein was prepared by removing water molecules, glycerol and ions from crystallization, keeping only the heme cofactor and the co-crystallized inhibitor NIH (2-{[3,5-bis(trifluoromethyl)benzyl]amino}-n-hydroxy-6-oxo-1,6-dihydropyrimidine-5-carboxamide). Docking parameters were determined based on an analysis of poses obtained by redocking, where the quality criterion was the root-mean-square deviation (RMSD) value. The best redocking solutions were identified by the lowest RMSD value. In this study, we have also evaluated the ChemScore, ChemPLP, and Goldscore scoring functions, varying the binding site radius by 10 and 20 Å around the reference atom, NE2 of His95 (ID 745). The ChemPLP function with a 10 Å radius around the reference atom produced a solution with an RMSD value of 0.47 Å compared to the crystallographic structure, thus being considered suitable parameters for the docking study of the molecules synthesized in this work. Ten runs of the genetic algorithm were performed for each molecule, and the figures presented in this work represent the best scores. GOLD (Jones et al. 1997) software v.2022.3.0 was used in docking studies. The visual inspection of intermolecular interactions and figures were generated using PyMOL v.2.4.0a0 (Schrödinger 2010) and Discovery Studio v.16.1.0.15350 (BIOVIA 2016).

#### Physicochemical and pharmacokinetic properties predictions

Physicochemical properties and pharmacokinetic parameters were predicted by using SwissADME server (Daina et al. 2017). The MPO inhibitor mitiperstat (AZD4831) was used as a control of these studies (Inghardt et al. 2022).

### Statistical analysis

All results were expressed as mean ± standard error of the mean. The statistical analyses were conducted using GraphPad Prism v.5.0 (GraphPad Software, USA). IC_50_, the concentration of the compounds required for 50% inhibition, was calculated using non-linear regression. For comparisons between groups, the One-way ANOVA test was used, followed by the Tukey post-test. P-values < 0.05 were considered significant.

## Results

### In vitro* pharmacological assays*

MPO activity was evaluated in the presence of other physiological substrates in addition to hydrogen peroxide and chloride. All phenyl hydrazide derivatives inhibited MPO chlorination activity. At 10 μM, **2a**, **2b**, **2e** and **2f** showed similar efficacy in inhibiting MPO activity (91.97 ± 0.3%; 86.87 ± 0.53%, 94.35 ± 0.22% and 88.4 ± 0.87%, respectively). On the other hand, **2c**, **2d** and **3** were less effective (77.71 ± 4.85%, 77.55 ± 0.47%, and 81.68 ± 0.39%, respectively; P < 0.05). The derivatives **2a**, **2b**, **2e** and **2f** were more potent than **2c**, **2d** and **3** (Table 1).

**Table 1:** Comparison of the pharmacological effects of compounds

Compound		MPO inhibition (IC_50,_ μM)	%SOD inhibition (50 μM)	%CAT inhibition (50 μM)	DPPH scavenging (IC_50,_ μM)	%Superoxide anion scavenging (100 μM)
2a	0.48 ± 0.03^*a*^		8.76 ± 5.66^*a*^	67.1 ± 3.6^*b*^	40.3 ± 4.36^*b*^	13.9 ± 6.79^*a*^
2b	0.36 ± 0.07^*a*^		−5.6 ± 3.73^*a*^	71.83 ± 2.5^*b,d*^	43.27 ± 3.43^*b*^	21.2 ± 8.03^*a,b*^
2c	1.89 ± 0.36^*b*^		8.5 ± 9.56^*a*^	68.03 ± 2.*7*^*b*^	ND	19.86 ± 4.*87*^*a*^
2d	2.57 ± 0.16^*c*^		−6.6 ± 11.39^*a*^	49.77 ± 1.9^*c*^	60.84 ± 2.82^*a*^	7.31 ± 3.93^*a*^
2e	0.34 ± 0.03^*a*^		−0.9 ± 11.39^*a*^	62.47 ± 5.*8*^*b,c,d*^	39.91 ± 3.21^*b*^	51.13 ± 9.44^*b*^
2f	0.27 ± 0.03^*a*^		8.37 ± 9.36^*a*^	77.92 ± 2^*d*^	42.61 ± 4.63^*b*^	22.69 ± 7.07^*a,b*^
3	1.67 ± 0.01^*b*^		−17.7 ± 2.04^*a*^	7.35 ± 1.*11*^*a*^	ND	11.63 ± 7.7^*a*^
DMSO	-		−7.2 ± 1.77^*a*^	1.95 ± 1.2^*a*^	-	−0.68 ± 1.57^*a*^
4-ABAH	0.22 ± 0.05^*a*^		-	-	-	-
Azide	-		-	97.81 ± 0.39^*e*^	-	-
Quercetin	-		-	-	12.8 ± 0.5^*c*^	90.79 ± 2.33^*c*^

Data are expressed as mean ± standard error of mean. Different letters (*a-e*) in the same column indicate statistically significant differences between groups (*p* < 0.05).

We also investigated the effect of the compounds on other enzymes, SOD and CAT, involved in the control of reactive species levels. At 50 µM, none of the derivatives significantly inhibited SOD activity. However, at the same concentration, **2a**, **2b**, **2c**, **2e** and **2f** reduced CAT activity by more than 60%. Compound **2d** was less effective, while **3** had no effect (Table 1).

The antioxidant activity of the derivatives was also studied by measuring the scavenging activity of the radicals DPPH and superoxide anion. At 200 μM, **2a**, **2b**, **2d**, **2e** and **2f** showed more than 90% DPPH scavenging activity, while **2c** showed a low efficacy in scavenging DPPH (24.64% ± 4%) and **3** was inactive. Compounds **2a**, **2b**, **2e** and **2f** showed comparable potency, with **2d** being the less potent. Only **2e** was able to scavenge superoxide anion, with an activity of 51.13 ± 9.44 at 100 µM (Table 1).

The structure–activity relationship analysis of this series of MPO inhibitors, as presented in Table 1, underscores the relevance of the open hydrazide scaffold for inhibitory efficacy. The structural cyclization to a quinazolinone core (compound **3**) resulted in a loss of potency, suggesting that the availability of the free hydrazide group for interaction with the heme moiety is a critical requirement.

Among the tested analogs, compounds **2a**, **2b**, **2e**, and **2f** exhibited equipotency to the reference standard 4-ABAH, with particular note to compound **2f**, which emerged as the most potent inhibitor. Electronic modulation of the aromatic system indicates that the introduction of a strong inductively electron-withdrawing group, such as fluorine in compound **2c**, resulted in decreased potency (1.89 ± 0.36 μM). This suggests that excessive depletion of electron density within the hydrazide moiety impairs its ability to coordinate with the heme iron or unfavorably alters the redox potential required for the inhibition mechanism. Conversely, the bromine atom in **2b**, while also electron-withdrawing, possesses lower electronegativity and higher lipophilicity—characteristics that preserved potency. Furthermore, the weak electron-donating methyl group (**2d**) failed to improve activity, likely due to *ortho* steric hindrance overriding any potential electronic benefit.

The lead compound **2f** demonstrates that the presence of electron-withdrawing groups (bromine) on both aromatic rings constitutes a beneficial substitution for activity (0.27 ± 0.03 μM). This effect is likely correlated with increased lipophilicity and adequate molecular volume to efficiently occupy the hydrophobic pockets of the active site.

### In silico* docking and pharmacokinetic predictions*

Since the substitutions at different positions of the compounds changed their potency for MPO inhibition, we also investigated the structure–activity relationship of the compounds by using molecular docking techniques. Molecular docking approaches have been extensively employed in several areas, especially in the study of protein–ligand (do Carmo Maquiaveli et al. 2023; Farias et al. 2021; Teixeira et al. 2022; Santos et al. 2020a, b) and protein–protein interactions (Farias et al. 2024; Durham et al. 2023), enabling the identification of binding sites and optimization of compounds capable of interacting with the enzyme's active site and inhibiting its catalytic activity.

**Fig. 3:**
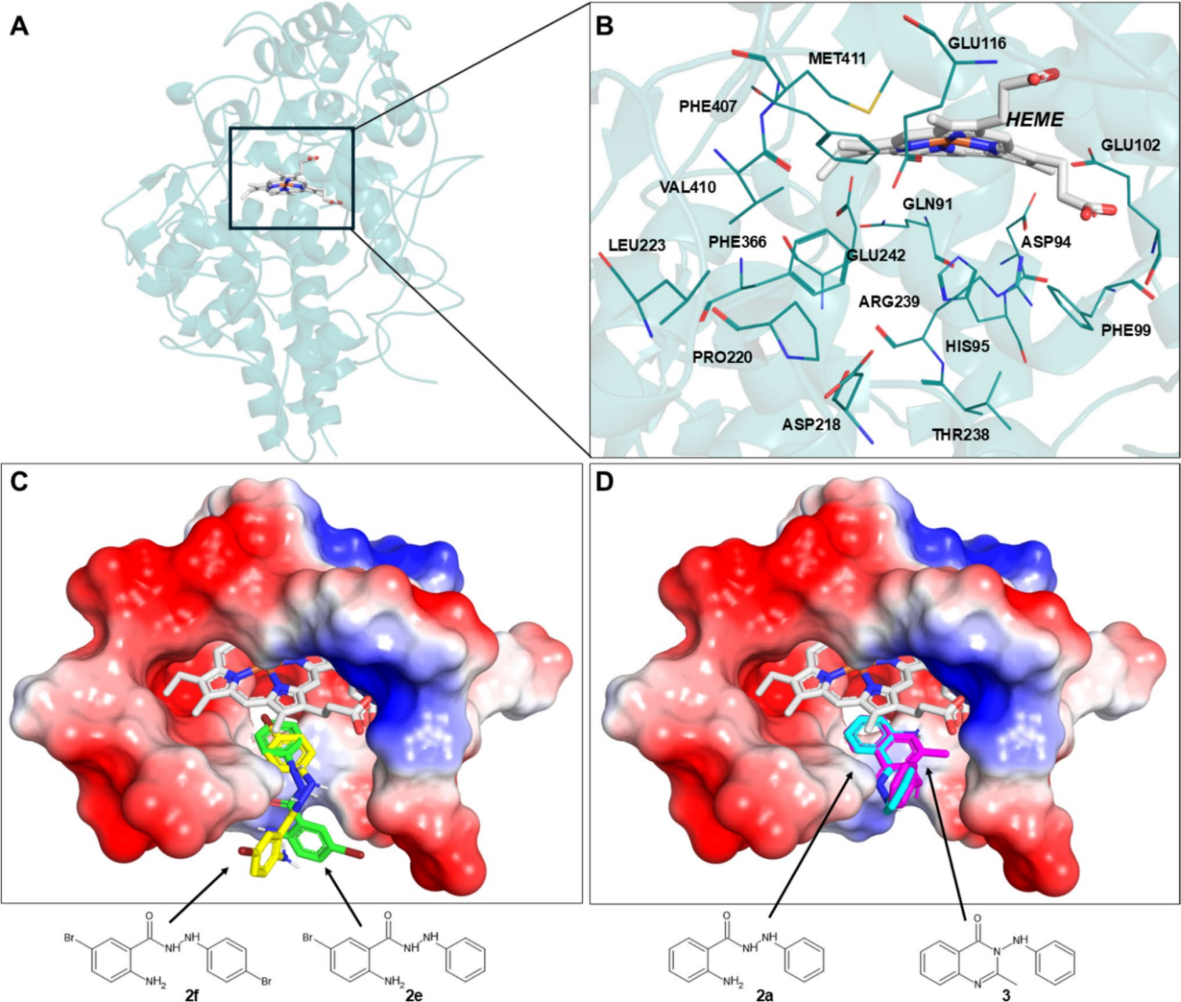
Predicted binding poses of selected phenylhydrazides within the binding site of MPO, obtained through molecular docking simulations. **A** Overview of MPO structure, highlighting the heme position; **B** Relevant amino acid residues around heme; **C** Binding poses of brominated inhibitors **2e** (green carbon atoms) and **2f** (yellow carbon atoms) within the MPO binding site and coloured according to electrostatic potential; **D** Comparison of **2a** (cyan carbon atoms) and its restricted analogue **3** (magenta carbon atoms) within the MPO cavity, with electrostatic potential colouring

Figure 3 shows the predicted binding modes for the studied molecules. Docking simulations allowed us to observe the location of the heme (Fig. 3a-b) and how the brominated (**2e** and **2f**) inhibitors interact with the cavity (Fig. 3c), which highlights a very similar orientation, in which the 2-amino-5-bromo phenyl ring of both compounds are oriented towards the bottom of the binding cavity, away from the heme group. In addition, the quinazolinone ring of (**3**) also exhibits a very similar orientation to the aniline ring of its analogue (**2a**) (Fig. 3d), although closer to the heme group. It's noteworthy that, although the hydrazide moiety does not exhibit significant interactions with the protein, it acts as a flexible linker between the two rings, allowing the inhibitors to have conformational freedom to adapt to the cavity.

**Fig. 4:**
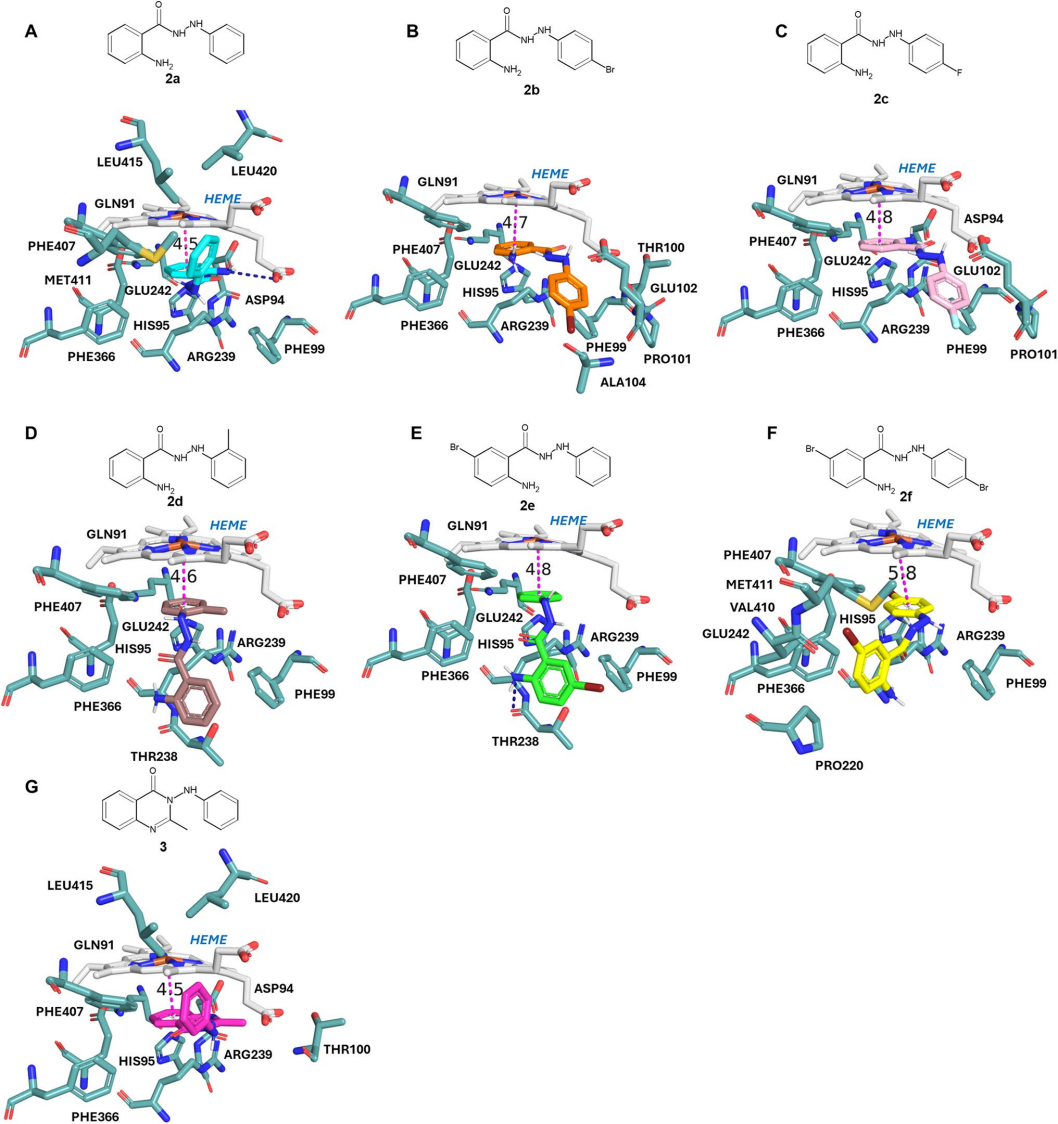
Interactions observed for ligands **2a** (**A**), **2b** (**B**), **2c** (**C**), **2d** (**D**), **2e** (E), **2f** (**F**), and **3** (**G**) within the MPO binding site. Heme-ring center distances (Å) are depicted in dashed magenta lines and hydrogen bonds in dark blue ones

Figure 4 illustrates the binding interactions between MPO and each compound, emphasizing the variety and magnitude of interactions that may be responsible for the enhancement of their inhibitory potency. This becomes evident when comparing molecules **2a** (Fig. 4A) and **2e** (Fig. 4E), where bromine induces a different binding orientation favouring π-π stacking with Phe99 and heme, as well as a hydrogen bond with Thr238. The relevance of bromine in the *meta* position of the phenyl ring of **2e** is highlighted by comparing it with molecule **2d** (Fig. 4D), where a very similar binding mode is observed, but loss of the interaction of the π-alkyl type with Phe99 is noted by the absence of bromine, resulting in a significant decrease in affinity. Conversely, including bromine in both rings (**2f**; Fig. 4F and S25, F) also leads to a distinct conformation, favouring halogen-bond type interactions with residues His90 and Met411 and while maintaining the π-π stacked interaction with heme, making it one of the most potent compounds in the series.

The docking studies revealed that replacing the bromine in the *para* position of the phenyl ring (Fig. 4B) for fluorine (Fig. 4C) resulted in a very similar conformation. However, the distance between heme and the 2-amino-5-bromo phenyl ring is longer in **2c** (4.7 *versus* 4.8 Å for **2b** and **2c**, respectively, Fig. 4B-C) while this ring and the *p*-Br-Ph ring in **2b** perform more VDW interactions, which suggests that bromine substitution enhances the binding affinity of the compound to the active site of MPO by a better fit to the binding cavity, thereby increasing its inhibitory potency (see Fig. S25 in Supporting Information).

Pharmacokinetics prediction using the SwissADME server revealed that none of the compounds violated Lipinski's or Veber's rules. Also, none of the compounds were predicted as glycoprotein P substrate. **2f** presented the worst CYP inhibitor profile, whereas **2c** does not appear to be a CYP inhibitor (Table 2).

**Table 2:** In silico pharmacokinetics analysis of *N*’-phenylbenzohydrazides **2a**-**f** and quinazolinone **3**

Molecule	2a	2b	2c	2d	2e	2f	3	Miperstat
MW (g/mol)	227.26	306.16	245.25	241.29	306.16	385.05	251.28	334.8
#Rotatable bonds	4	4	4	4	4	4	2	3
#H-bond acceptors	1	1	2	1	1	1	2	3
#H-bond donors	3	3	3	3	3	3	1	3
TPSA (Å^2^)	67.15	67.15	67.15	67.15	67.15	67.15	46.92	102.48
Consensus Log P	1.93	2.54	2.19	2.39	2.56	3.25	2.69	0.84
GI absorption	High	High	High	High	High	High	High	High
BBB permeant	Yes	Yes	Yes	Yes	Yes	Yes	Yes	No
Pgp substrate	No	No	No	No	No	No	No	Yes
CYP1A2 inhibitor	No	No	No	No	No	Yes	Yes	No
CYP2C19 inhibitor	No	Yes	No	Yes	Yes	Yes	Yes	No
CYP2C9 inhibitor	Yes	Yes	No	Yes	Yes	Yes	No	No
CYP2D6 inhibitor	No	Yes	No	Yes	Yes	Yes	No	No
CYP3A4 inhibitor	No	No	No	No	No	Yes	No	No
Lipinski #violations	0	0	0	0	0	0	0	0
Veber #violations	0	0	0	0	0	0	0	0

## Discussion

The current study demonstrates that the phenyl hydrazide-based compounds tested are strong MPO inhibitors. Blocking MPO's chlorination activity might help explain the anti-inflammatory effects of **2a-d** in the carrageenan-induced subcutaneous air pouch mice model, where these compounds reduced cell migration, cytokine production, and protein leakage (Paiva et al. 2022).

MPO can oxidize multiple substrates due to the relatively high reduction potentials of the Compound I/native enzyme (1.16 V), Compound I/Compound II (1.35 V) and Compound II/native enzyme (0.97 V). When inhibitors are poorly oxidized by Compound II, they cause its accumulation, decreasing HOCl production (Jantschko et al. 2005; Bensalem et al. 2014). These inhibitor types are not effective in physiologic environments because there are many physiological reductants that can enhance MPO catalytic cycle by acting as substrates for Compound II (Forbes and Kettle 2018). Considering that phenyl hydrazides were effective in inhibiting MPO chlorination activity in the presence of physiological reductants, we can suggest that they are physiologically effective inhibitors.

The unsubstituted phenyl rings and phenyl rings bearing bromine led to the most potent compounds in the series. The comparison of our results with a recent work utilizing acylhydrazone derivatives (Santos et al. 2020a, b) emphasizes the consistency of our findings, since the substance with bromine in the *meta* position of the phenyl ring resulted in 90% of MPO inhibition at 10 μM, while substitutions with fluorine or chlorine did not, supporting the notion that bromine substitution plays a key role in enhancing the inhibitory activity of the compounds. Overall, these findings provide valuable insights into the structure–activity relationship of the synthesized compounds and their potential as MPO inhibitors.

Since MPO is a metalloenzyme that can be inhibited through binding to the iron of the heme group present in the enzyme's catalytic site, we also investigated the effect of the compounds on the metalloenzymes SOD and CAT. None of the compounds inhibited SOD, indicating that the compounds do not inhibit superoxide anion formation. Although the activity of the antioxidant enzyme CAT was inhibited by some compounds, it is important to highlight that the inhibition of CAT was observed in higher concentrations than those observed for MPO inhibition. For example, derivative **2d** was the least potent in inhibiting MPO and it only inhibited 50% of CAT activity at a concentration 20 times higher.

Regarding the antioxidant activity through radical scavenging, it was observed that the different substitutions impacted this activity. The loss of the -NH_2_ group of aniline impaired the ability of **3** to react with DPPH since it was inactive. The methyl substitution in the *ortho* position of the phenyl and other substituents in the *para* position in **2d** seem to contribute to its decreased potency through steric and electronic factors. In the same way, the high electronegativity of the fluorine substituent in **2c** appears to reduce its potency for DPPH scavenging activity. For superoxide anion scavenge, only **2e**, which presents a bromine substituent in the aniline ring, was active. Interestingly, the addition of a bromine substituent in the phenyl ring, as in **2f**, resulted in loss of activity.

All compounds showed high predicted gastrointestinal absorption, which is in accordance with the previously reported in vivo anti-inflammatory effect observed for **2a-d** after oral administration (Paiva et al. 2022). In addition, the predicted blood–brain barrier penetration is an interesting characteristic, considering that MPO plays a role in the physiopathology of inflammatory diseases that affect the central nervous system (Chen et al. 2024; Smyth et al. 2022; Boonpraman et al. 2023).

Despite the promising results, this study has limitations. Cell-free in vitro assays did not assess cytotoxicity or selectivity like cellular models, and the predicted pharmacokinetic parameters and docking interactions require experimental validation. Further studies with in vivo models of MPO-driven inflammation and metabolic stability assays are necessary to confirm the potential of these compounds.

Taken together, our findings showed that *N*’-phenylbenzohydrazide compounds are potent MPO inhibitors, indicating their pharmacological potential to reduce oxidative stress by impairing MPO-induced formation of reactive species, primarily HOCl. The inhibitory activity of the derivatives against MPO was generally maintained despite structural variations. In contrast, their radical scavenging activity appeared more sensitive to substituent changes. Taken together, these findings provide initial insights into the structural features of N′-phenylbenzohydrazides that may influence binding to the MPO catalytic site. These results could contribute to the rational design and optimisation of future MPO inhibitors.

## Supplementary Information

Below is the link to the electronic supplementary material.

Supplementary file1 (DOCX 2022 KB)

## Data Availability

The authors declare that, should any raw data files be required in an alternative format, these are available from the corresponding author upon reasonable request.
