# Supplementary material for: Evaluation of phenyl hydrazide-based compounds as myeloperoxidase inhibitors
Source: Naunyn Schmiedebergs Arch Pharmacol. 2026 Jan 29;399(7):10087–96. doi: 10.1007/s00210-025-04926-x (PMC13152889; doi:10.1007/s00210-025-04926-x)
Supplement: Supplementary file 1 — Supplementary file1 (DOCX 2022 KB) [file 210_2025_4926_MOESM1_ESM.docx]

**Supplementary Information**

**Evaluation of Phenyl Hydrazide-based Compounds as Myeloperoxidase Inhibitors**

***Laryssa C. C. L. Salema,^a^ André B. Farias,^b,c^ Tiago R. Navarro,^d^ Patrick P.***

***Pimentel,^d,e^ Thuany B. S. Aguiar,^d^ Nelilma C. Romeiro,^b^ Evanoel C. de Lima,^d^ Juliana***

***M. Raimundo,^a^ Leandro L. da Silva^*a^***

*^a^Grupo de Pesquisa em Farmacologia de Produtos Bioativos, Centro Multidisciplinar UFRJ-Macaé, Universidade Federal do Rio de Janeiro, Av.Aluízio da Silva Gomes 50, 27930-560, Macaé, RJ, Brazil*

^b^Laboratório Integrado de Computação Científica (LICC), Centro Multidisciplinar UFRJ-Macaé, Universidade Federal do Rio de Janeiro, Av. Aluízio da Silva Gomes 50, 27930-560, Macaé, RJ, Brazil.

^c^Laboratório Nacional de Computação Científica—LNCC, Av. Getúlio Vargas 333, 25651-075, Petrópolis, RJ, Brazil.

*^d^Laboratório de Catálise e Síntese de Substâncias Bioativas, Instituto Multidisciplinar de Química, Centro Multidisciplinar UFRJ-Macaé, Universidade Federal do Rio de Janeiro, Estrada do Imburo s/n, 27971-525, Macaé, RJ, Brazil.*

*^e^Laboratório de Catálise e Síntese (LabCSI) - Campus Valonguinho - Universidade Federal Fluminense, 24020-140, Niteroi, RJ, Brazil.*

**2-amino-N'-phenylbenzohydrazide (2a)**

Yield, 80%. ^1^H NMR (DMSO-d6, 400 MHz) d ppm 10.06 (s, 1H), 7.76 (s, 1H), 7.66 (d, 1H, J = 7.8), 7.20–7.12 (m, 3H), 6.77 (d, 2H, J = 7.7), 6.73–6.68 (m, 2H), 6.54 (t, 1H, J = 7.8), 6.36 (s, 2H). MS (EI): m/z 227 (22%), m/z 120 (100%), m/z 92 (25%), m/z 121 (8%), and m/z 77 (8%).


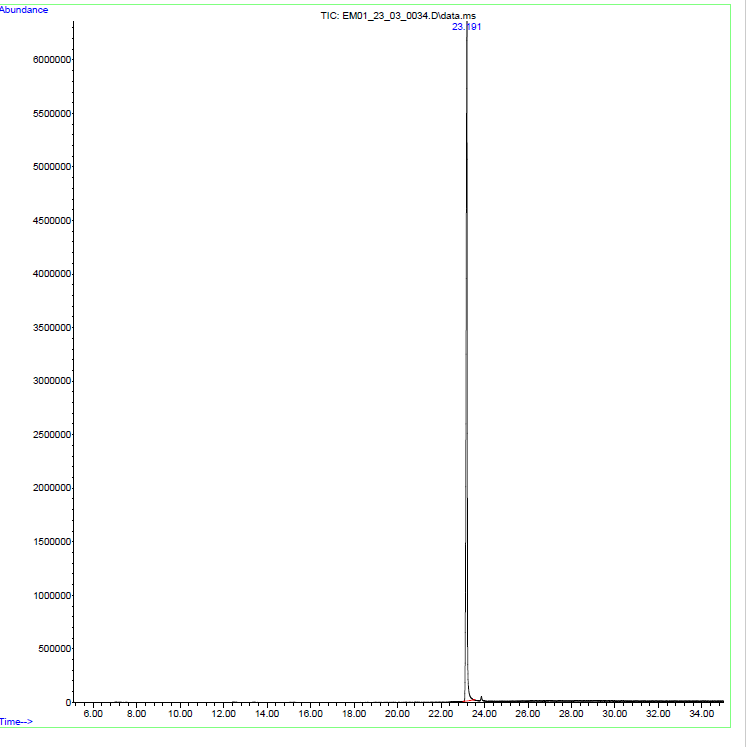


**Fig. S1** Gas chromatography spectrum of compound **2a**


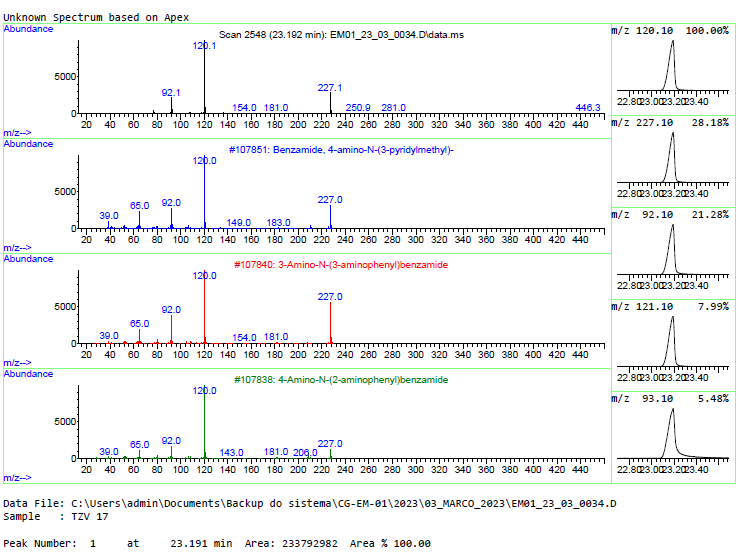


**Fig. S2** Mass spectrum of compound **2a**


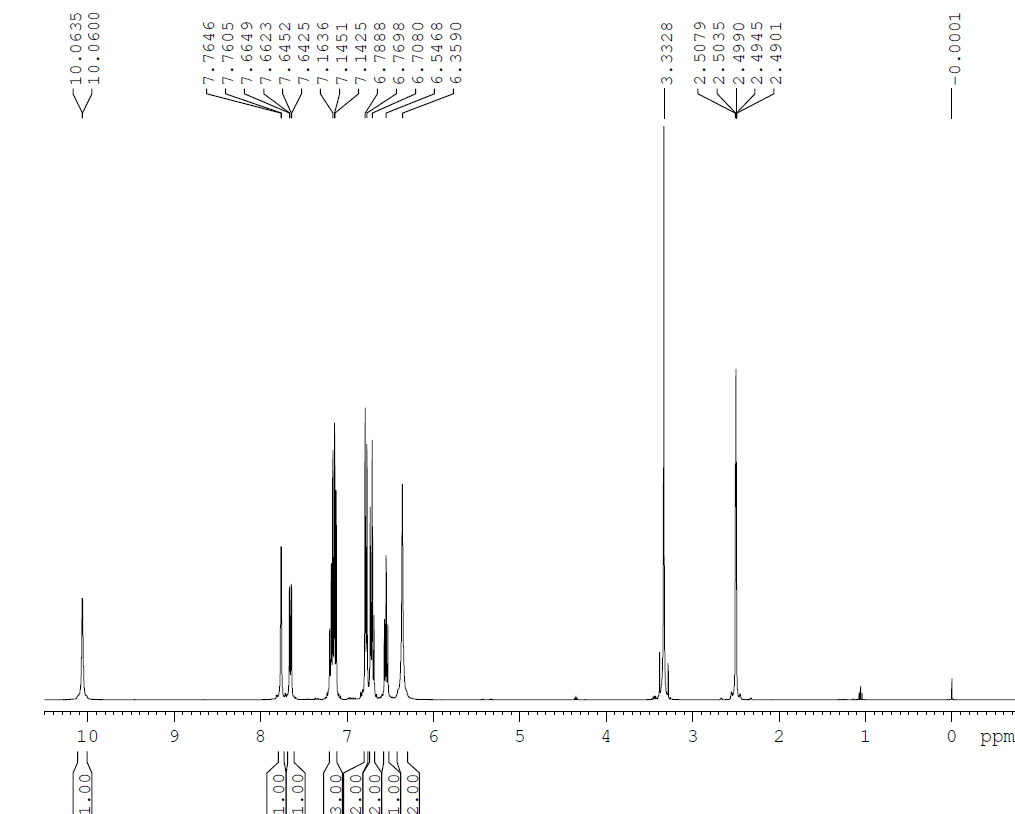


**Fig. S3** ^1^H NMR spectrum (400 MHz, DMSO-*d*_6_) of compound **2a**


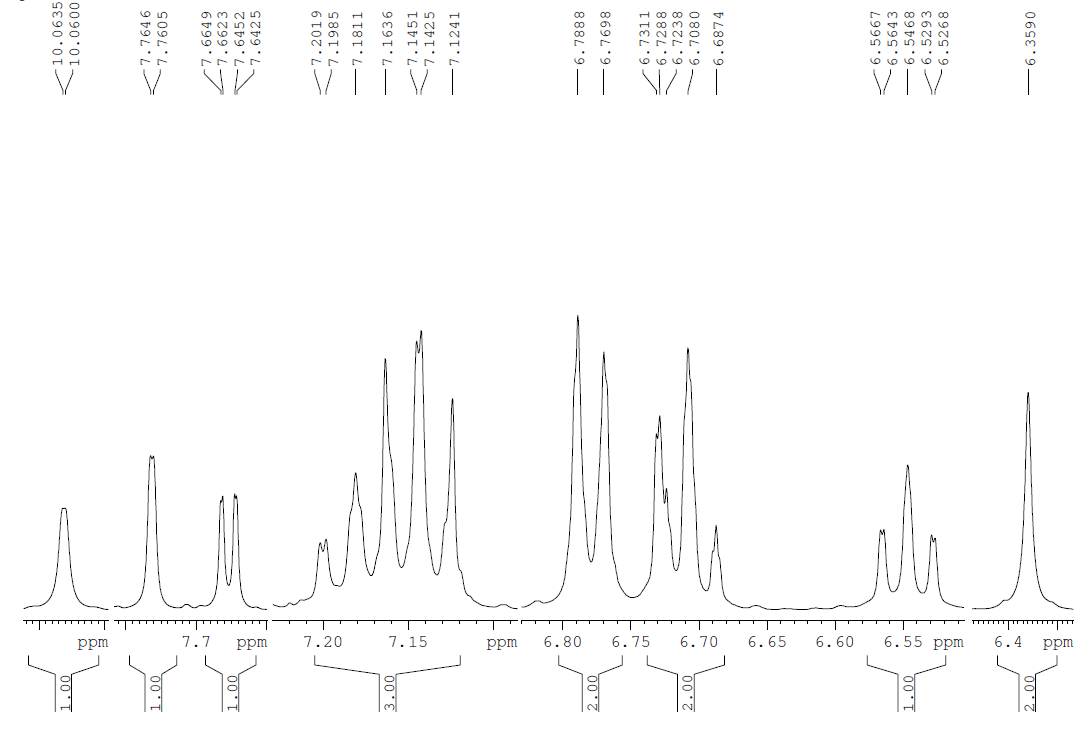


**Fig S4** ^1^H NMR spectrum (400 MHz, DMSO-*d*_6_) of compound **2a**

**2-amino-N'-(4-bromophenyl)benzohydrazide (2b)**

Yield, 61%. ^1^H NMR (DMSO-d6, 400 MHz) d ppm 10.14 (s, 1H), 8.01 (s, 1H), 7.65 (d, 1H, J = 7.9Hz), 7.29 (d, 2H, J = 7.9Hz), 7.18 (t, 1H, J = 8.9 Hz), 6.73 (d, 3H, J = 8.0 Hz), 6.54 (t, 1H, J = 8.9 Hz), and 6.41 (s, 2H). MS (EI): m/z 305 (9%), m/z 307 (9%), m/z 120 (100%), m/z 92 (23%), and m/z 121 (8%).


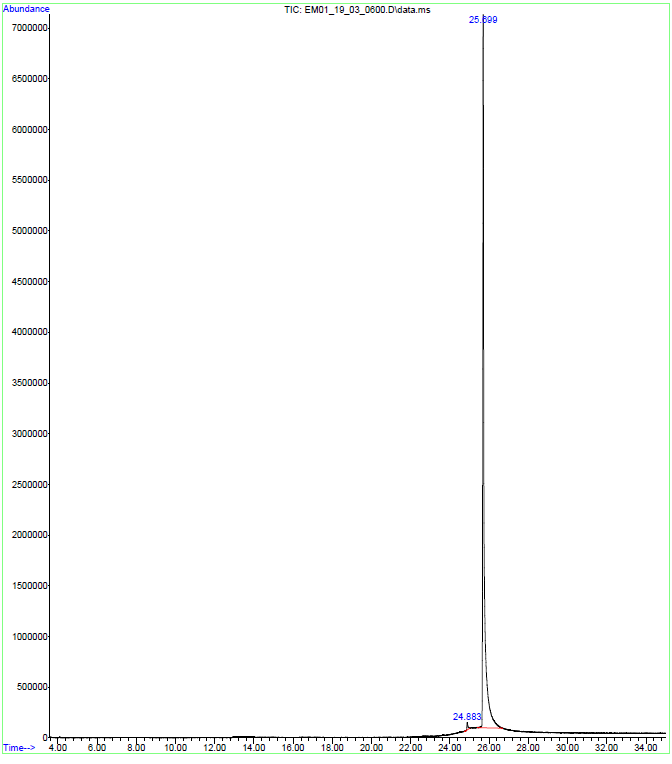


**Fig. S5** Gas chromatography spectrum of compound **2b**


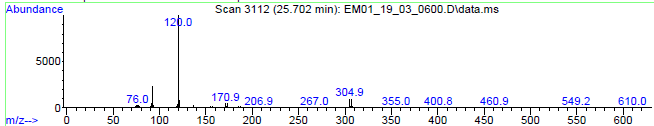


**Fig. S6** Mass spectrum of compound **2b**


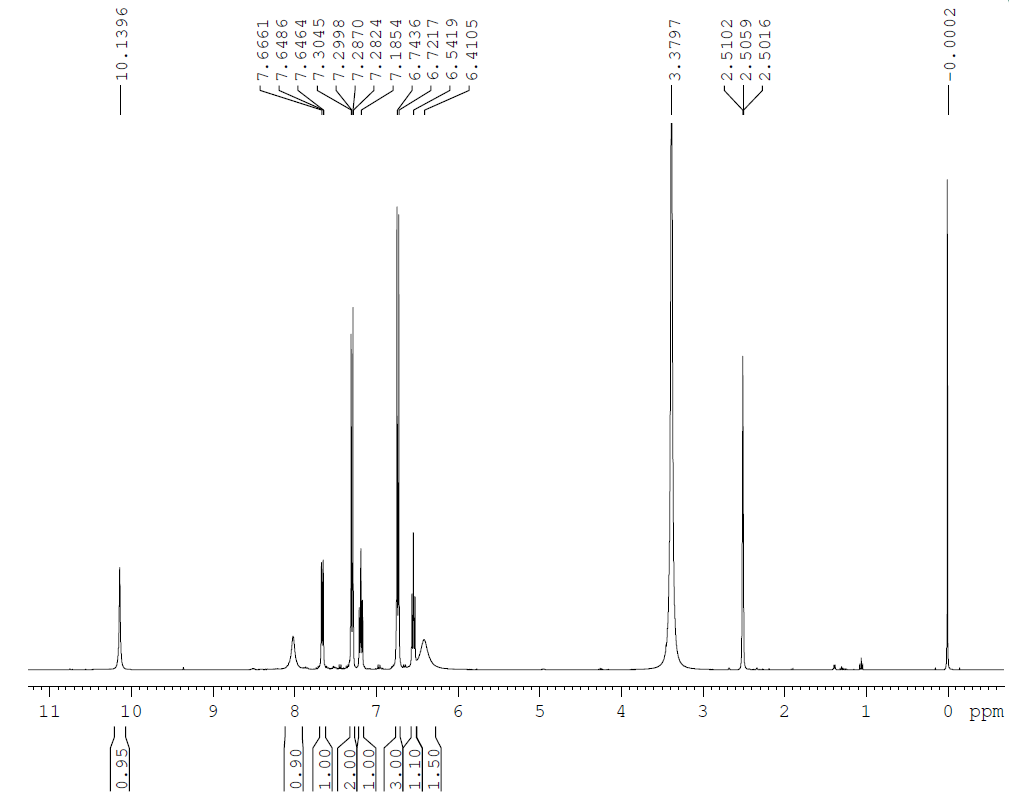


**Fig. S7** ^1^H NMR spectrum (400 MHz, DMSO-*d*_6_) of compound **2b**


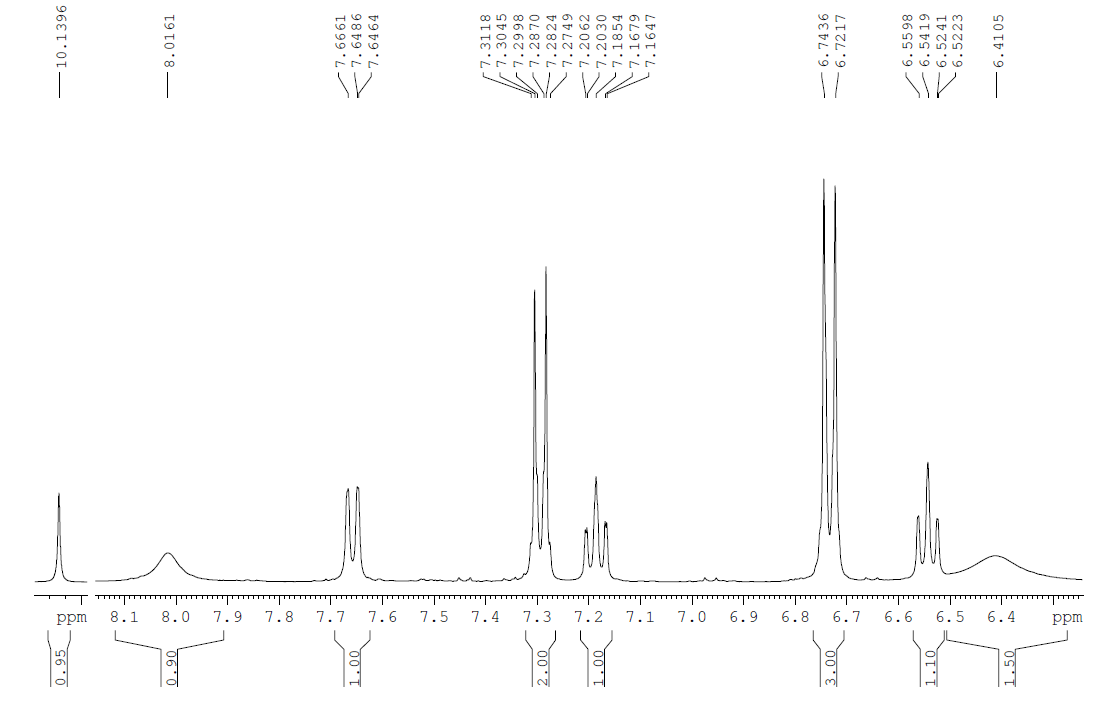


**Fig. S8** ^1^H NMR spectrum (400 MHz, DMSO-*d*_6_) of compound **2b**

**2-amino-N'-(4-fluorophenyl)benzohydrazide (2c)**

Yield, 45%. ^1^H NMR (DMSO-d6, 400 MHz) d ppm 10.12 (s, 1H), 7.76 (s, 1H), 7.65 (d, 1H, J = 7.9 Hz), 7.18 (t, 1H, J = 8.3 Hz), 6.99 (t, 2H, J = 7,0 Hz), 6.80–6.76 (m, 2H), 6.72 (d, 1H, J = 8.2 Hz), 6.53 (t, 1H, J = 7.8 Hz), and 6.38 (s, 2H). MS (EI): m/z 245 (21%), m/z 246 (3%), m/z 120 (100%), m/z 92 (20%), and m/z 121 (8%).


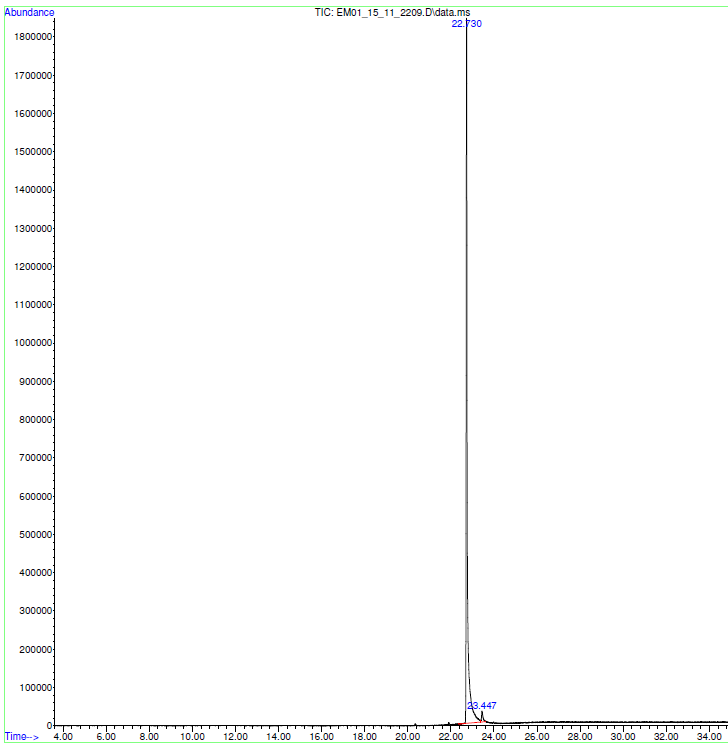


**Fig. S9** Gas chromatography spectrum of compound **2c**


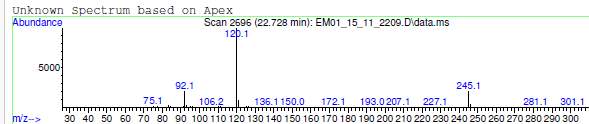


**Fig. S10** Mass spectrum of compound **2c**


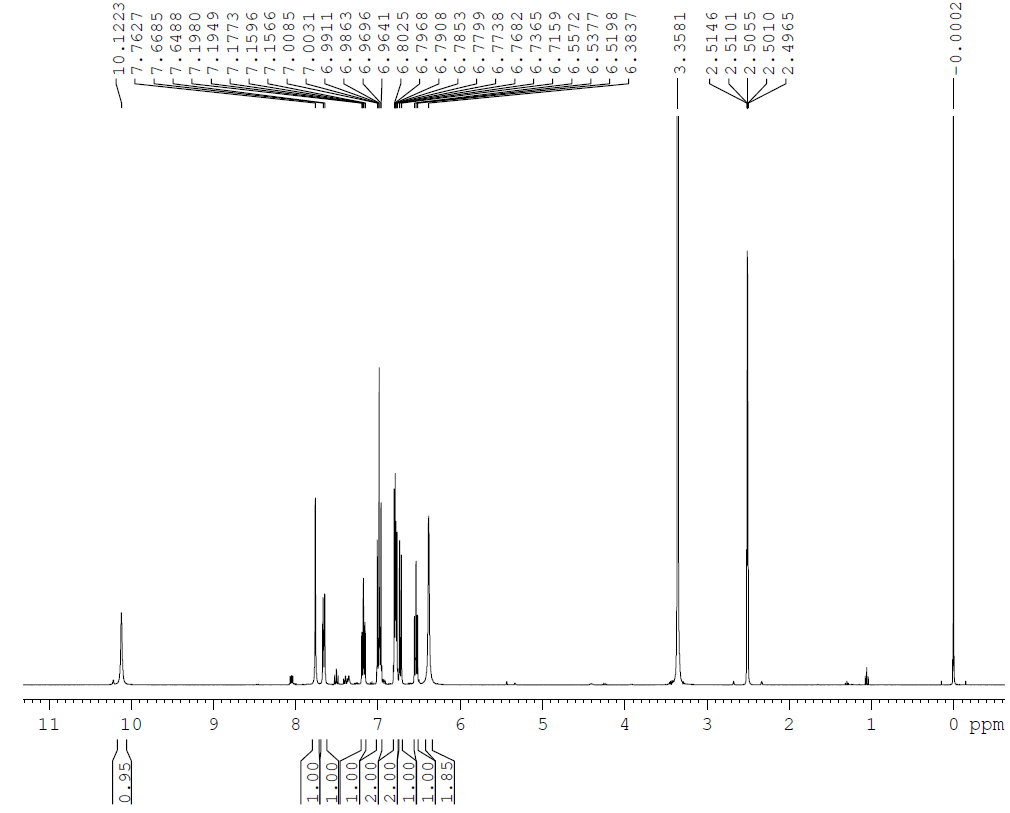


**Fig. S11** ^1^H NMR spectrum (400 MHz, DMSO-*d*_6_) of compound **2c**


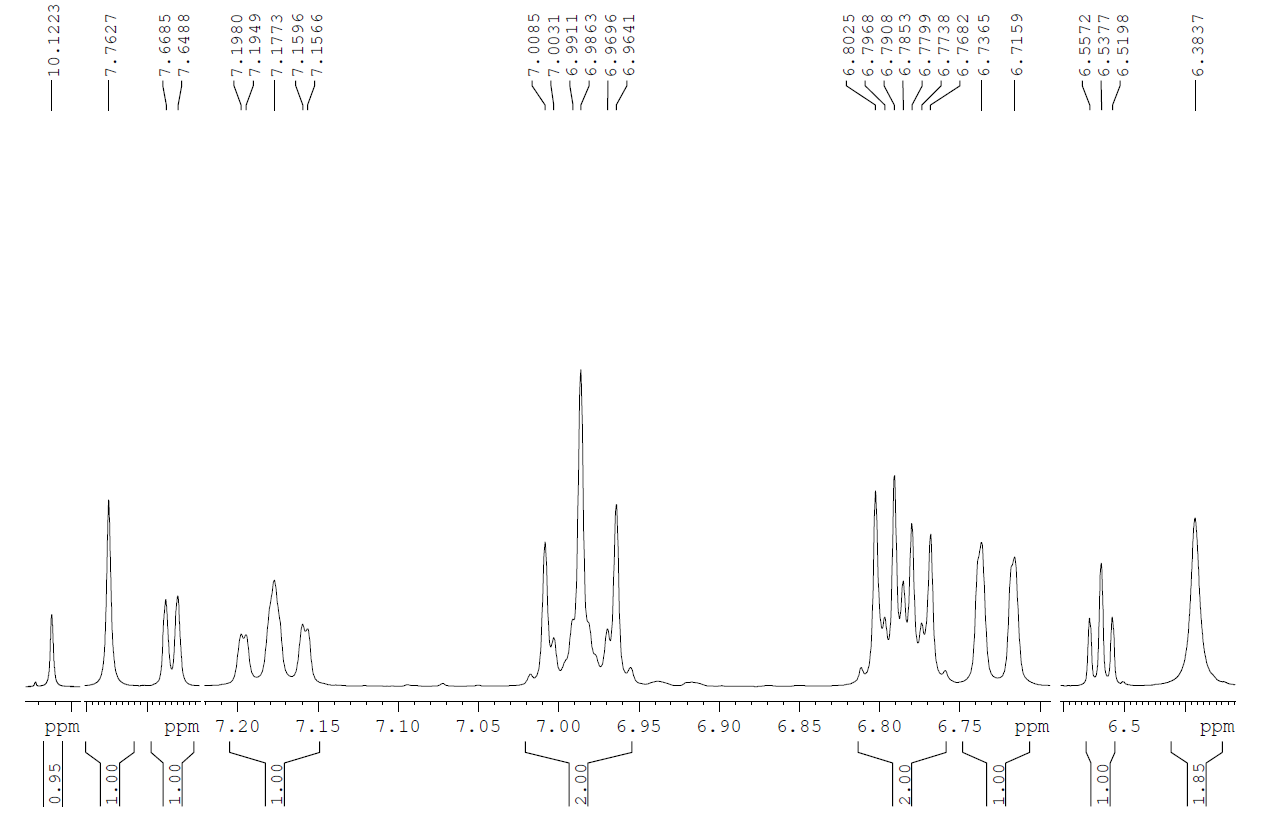


**Fig. S12** ^1^H NMR spectrum (400 MHz, DMSO-*d*_6_) of compound **2c**

**2-amino-N'-(*o*-tolyl)benzohydrazide (2d)**

Yield, 78%. ^1^H NMR (DMSO-d6, 400 MHz) d ppm 10.13 (s, 1H), 6.3 (d, 1H, J = 7.9 Hz), 7.18 (t, 1H, J = 6.6 Hz), 7.13 (s, 1H), 7.02–6.99 (m, 2H), 6.73 (d, 1H, J = 6.7 Hz), 6.71 (d, 1H, J = 6.3 Hz), 6.67 (t, 1H, J = 6.4 Hz), 6.55 (t, 1H, J = 6.3 Hz), 6.38 (s, 2H), and 2.21 (s, 3H). MS (EI): m/z 241 (26%), m/z 120 (100%), m/z 92 (18%), m/z 106 (5%), and m/z 121 (8%).


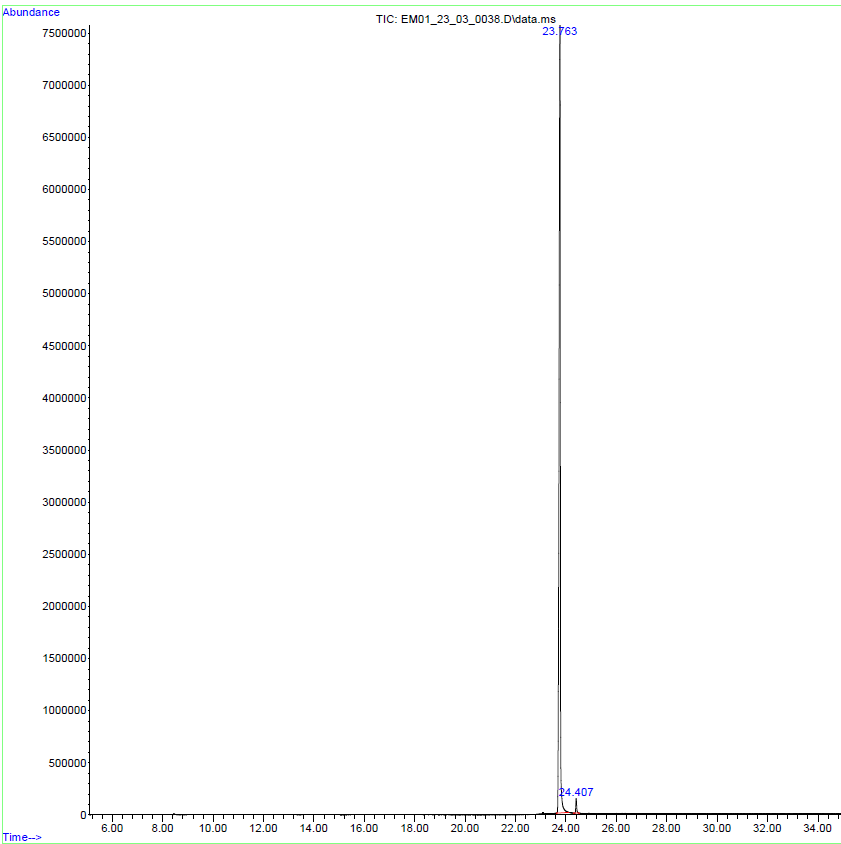


**Fig. S13** Gas chromatography spectrum of compound **2d**


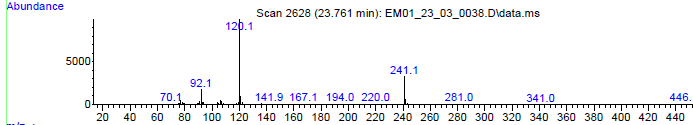


**Fig. S14** Mass spectrum of compound **2d**


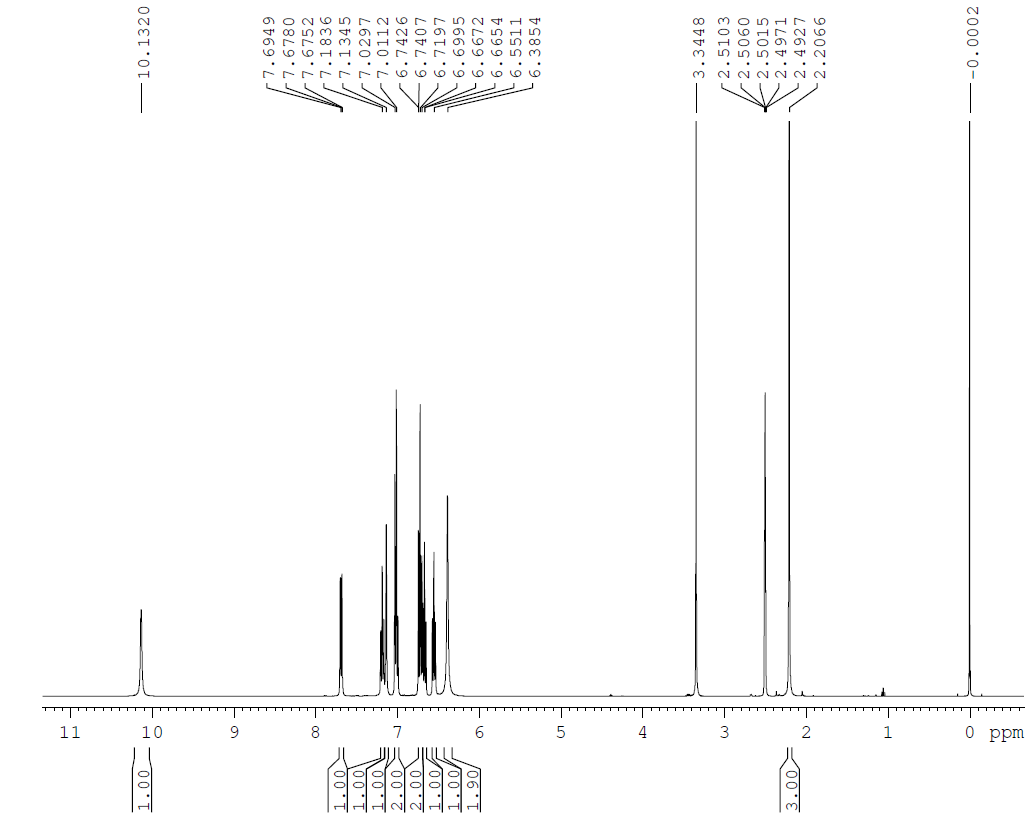


**Fig. S15** ^1^H NMR spectrum (400 MHz, DMSO-*d*_6_) of compound **2d**

**2-amino-5-bromo-N’-phenylbenzohydrazide (2e)**

Yield, 55%. ^1^H NMR (DMSO-d6, 400 MHz) d ppm 10.19 (s, 1H), 7.80 (d, 2H, J = 7.6 Hz), 7.32 (d, 1H, J = 7.6 Hz), 7.15 (t, 2H, J = 7.5 Hz), 6.77 (d, 2H, J = 7.7 Hz), 6.73-6.69 (m, 2H, J = 7.3 Hz), and 6.54 (s, 2H). MS (EI): m/z 307 (34%), m/z 305 (34%), m/z 200 (100%), m/z 198 (99%), m/z 170 (21%).


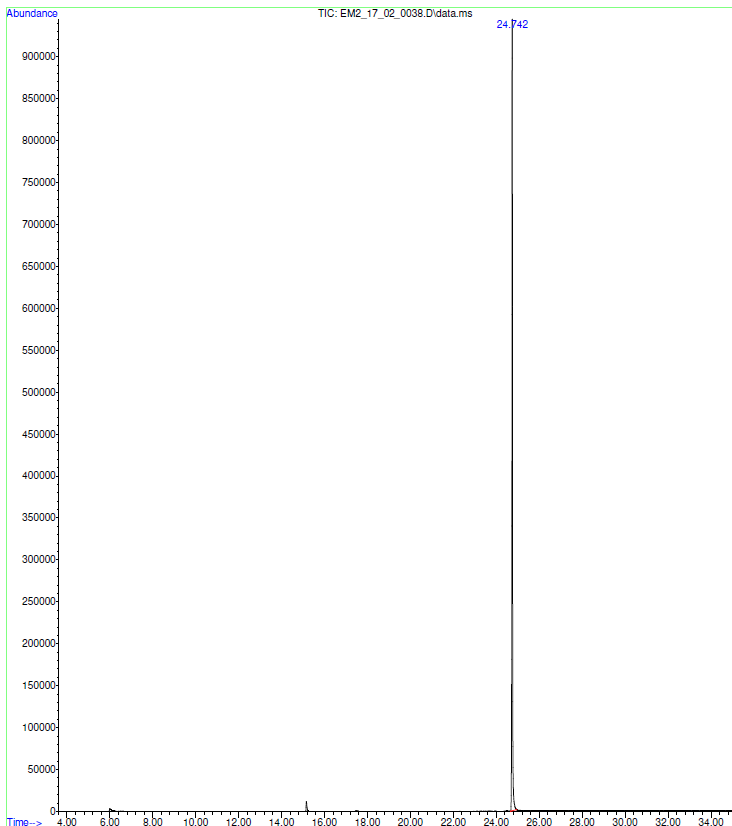


**Fig. S16** Gas chromatography spectrum of compound **2e**


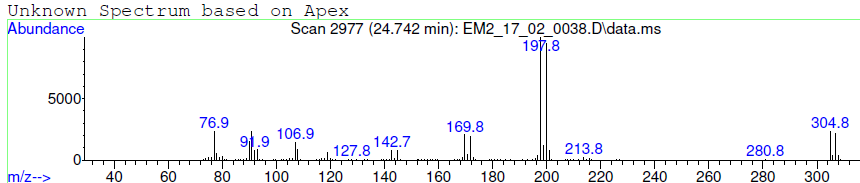


**Fig. S17** Mass spectrum of compound **2e**


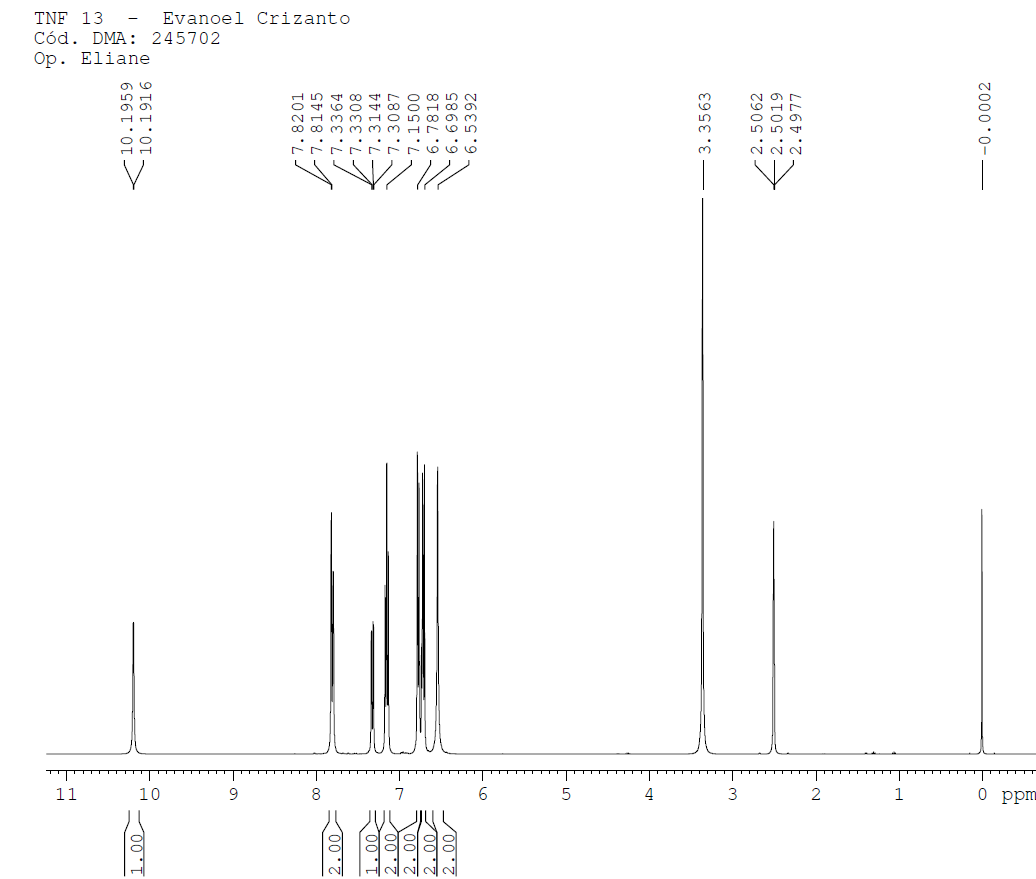


**Fig. S18** ^1^H NMR spectrum (400 MHz, DMSO-*d*_6_) of compound **2e**

**2-amino-5-bromo-N’-(4-bromophenyl) benzohydrazide (2f)**

Yield, 52%. ^1^H NMR (DMSO-d6, 400 MHz) d ppm 10.21 (s, 1H), 7.99 (s, 1H), 7.80 (d, 1H, *J* = 2.3 Hz), 7.31-7.28 (m, 3H), 6.74-6.70 (m, 3H), and 6.54 (s, 2H). MS (EI): m/z 385 (22%), m/z 200 (98%), m/z 198 (100%), m/z 172 (16%), m/z 170 (16%).


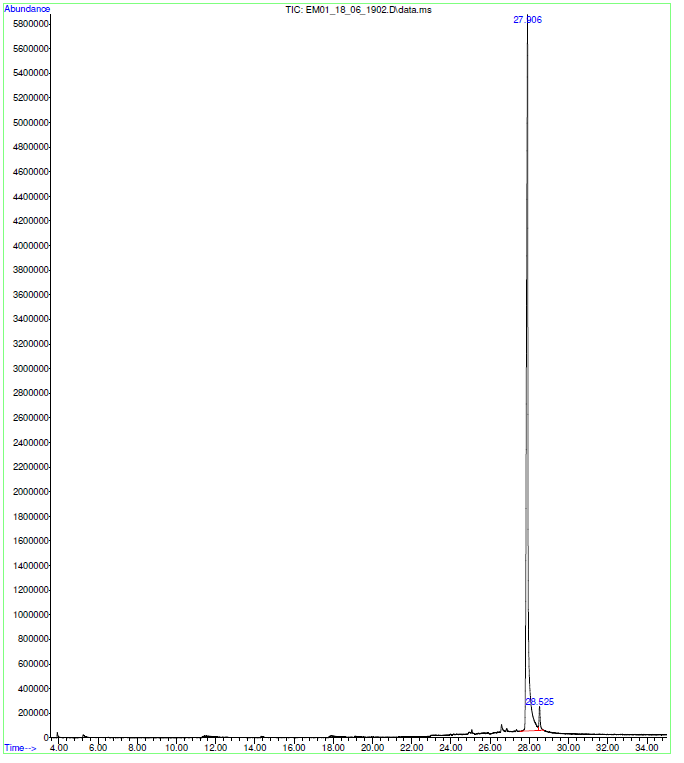


**Fig. S19** Gas chromatography spectrum of compound **2f**


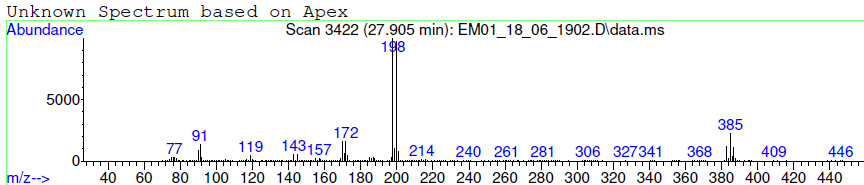


**Fig. S20** Mass spectrum of compound **2f**


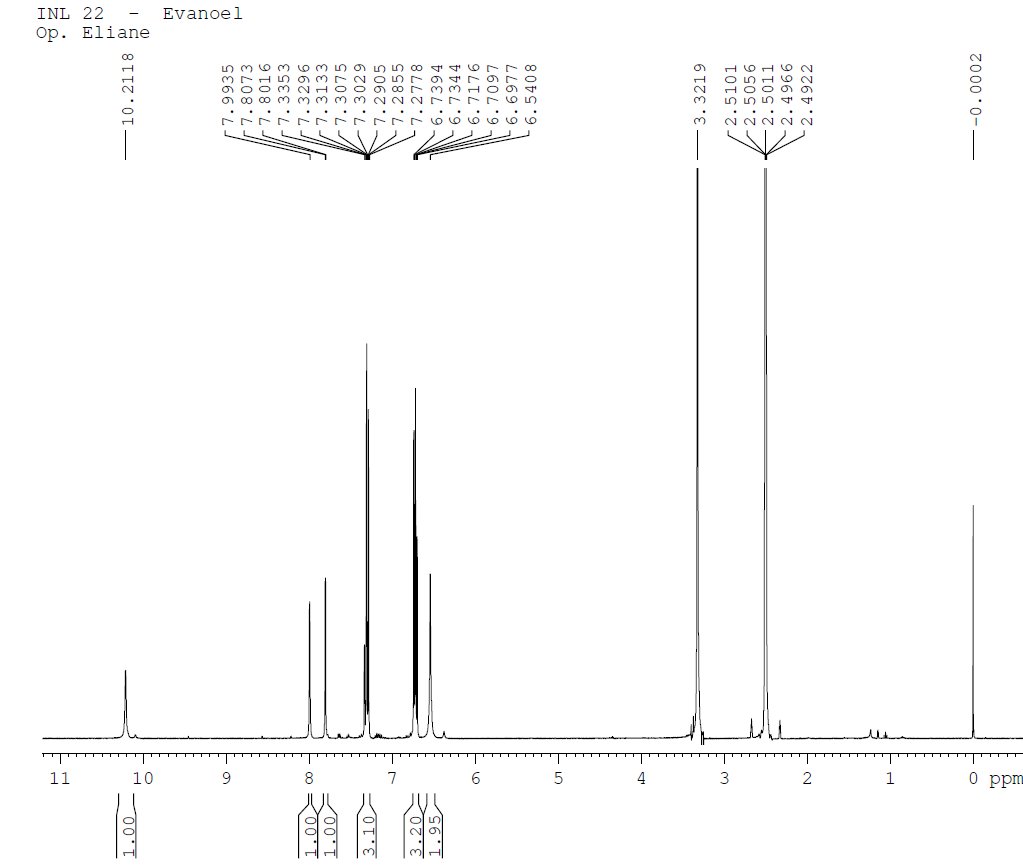


**Fig. S21** ^1^H NMR spectrum (400 MHz, DMSO-*d*_6_) of compound **2f**


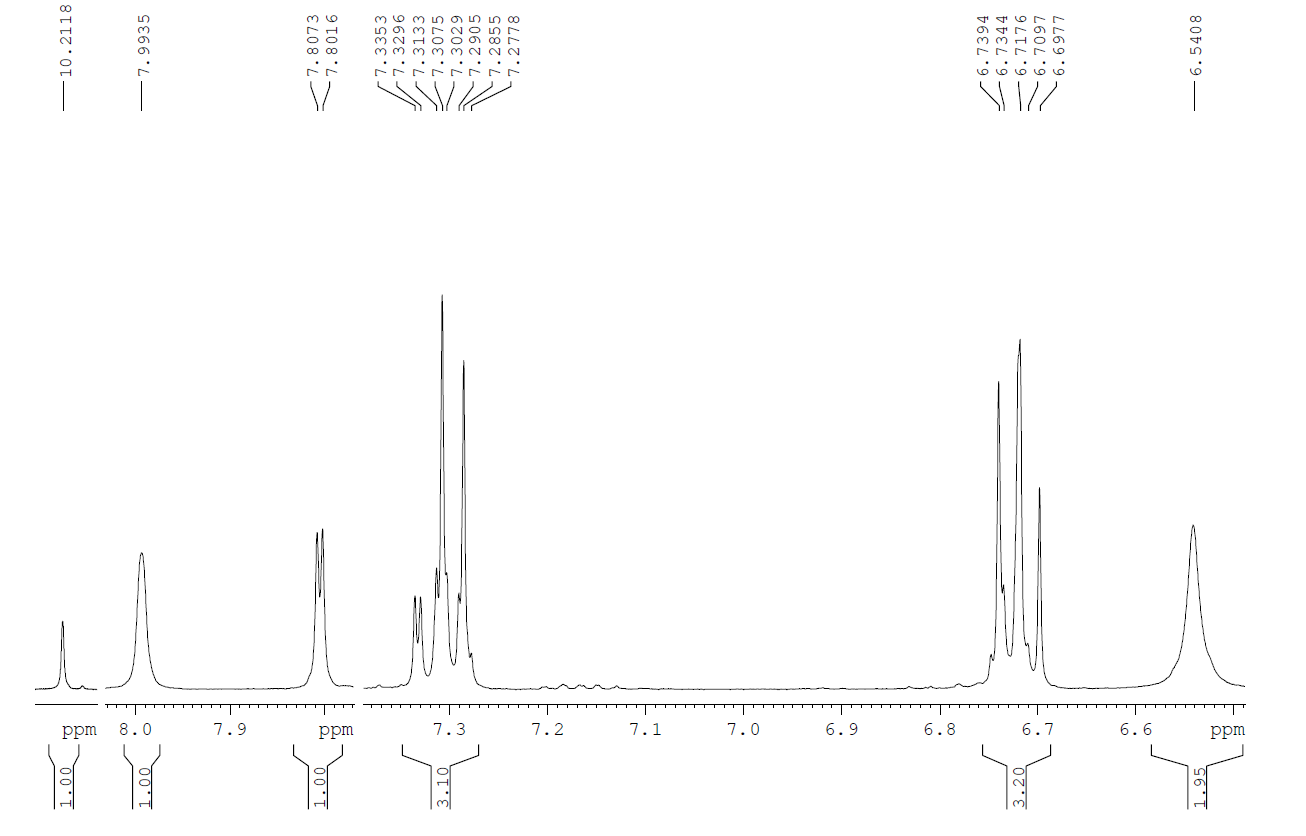


**Fig. S22** ^1^H NMR spectrum (400 MHz, DMSO-*d*_6_) of compound **2f**

**2-methyl-3-(phenylamino)quinazolin-4(*3H*)-one (3)**

Yield, 85%. ^1^H NMR (DMSO-d6, 400 MHz) d ppm 8.36 (d, 1H, J = 7.8 Hz), 8.04 (t, 1H, J = 7.6 Hz), 7.81-7.74 (m, 4H), 7.54 (d, 3H, J = 7.6 Hz), and 2.24 (s, 3H). MS (EI): m/z 146 (37%), m/z 77 (18%), m/z 146 (37%), m/z 117 (17%), m/z 252 (17%).


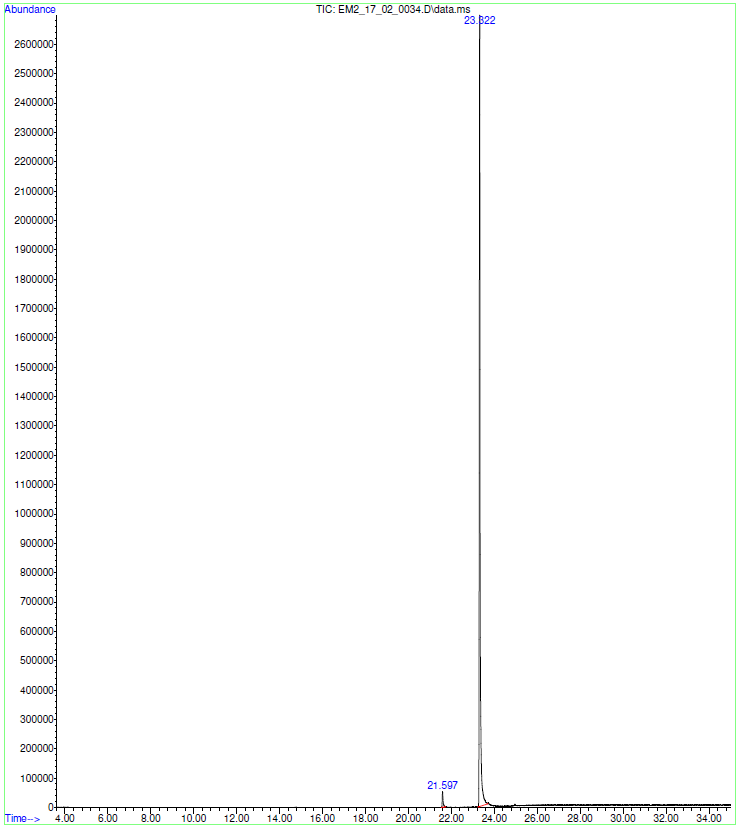


**Fig. S23** Gas chromatography spectrum of compound **3**


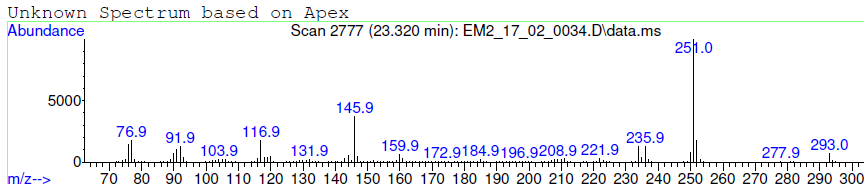


**Fig. S24** Mass spectrum of compound **3**


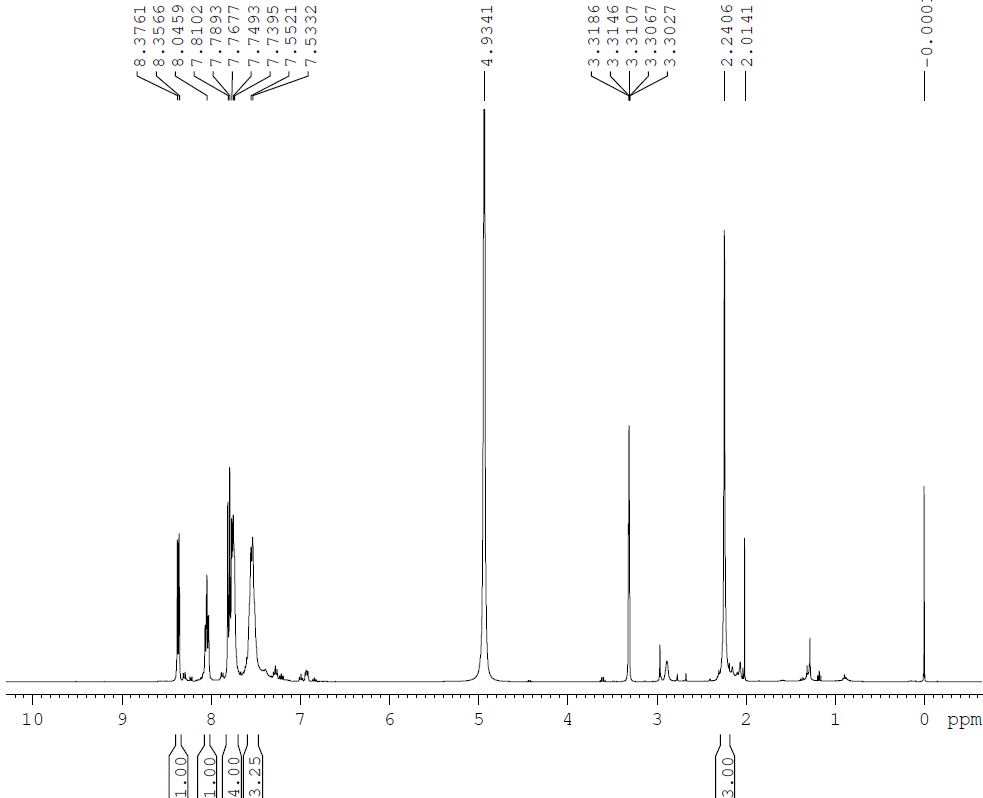


**Evaluation of N'-phenylbenzohydrazide-based compounds as myeloperoxidase inhibitors**


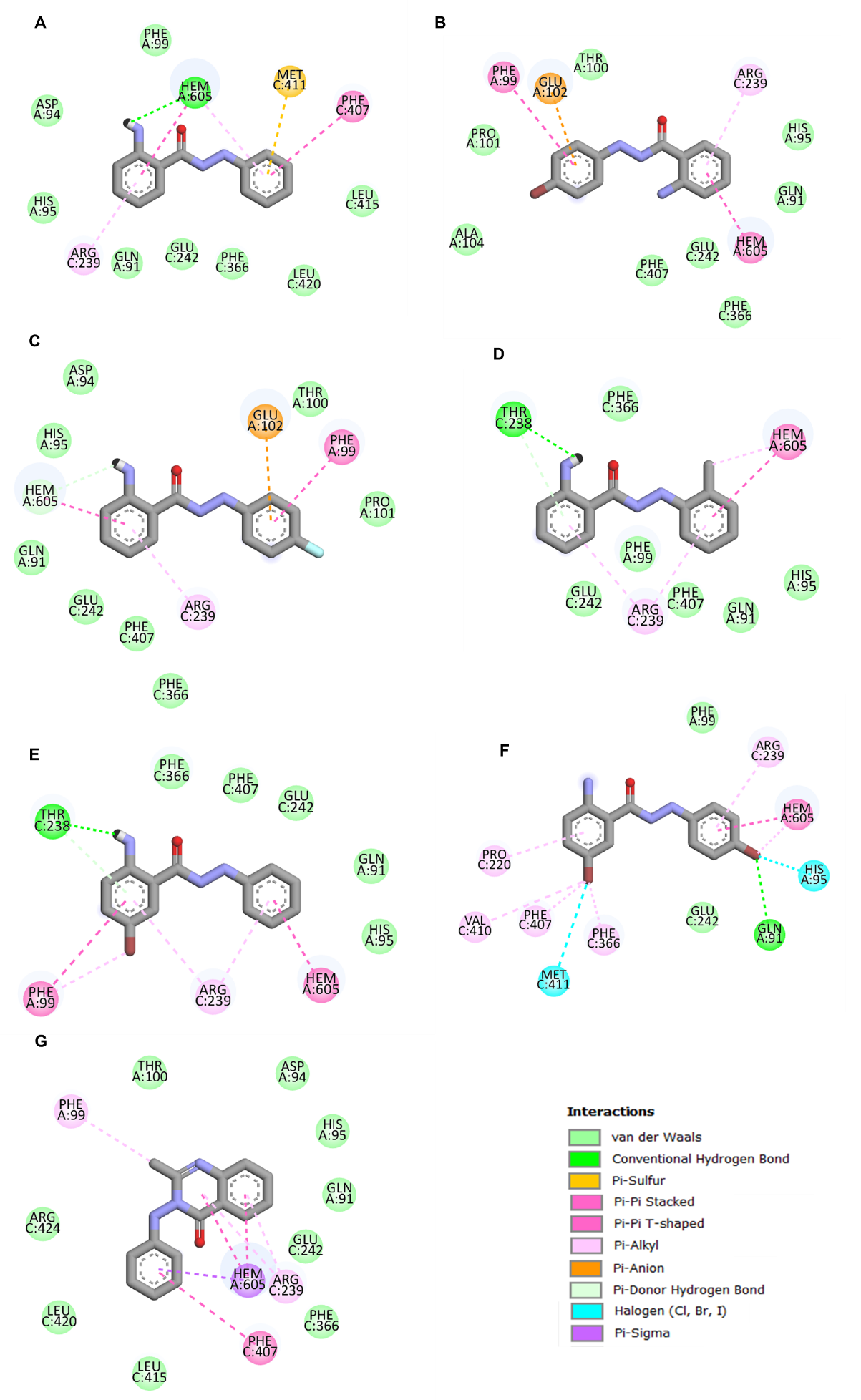


**Fig. S25** Analysis of the molecular interactions between the compounds 2a (**A**), 2b (**B**), 2c (**C**), 2d (**D**), 2e (**E**), 2f (**F**) and 3 (**G**) obtained by the molecular docking study. Interaction types were identified using the DiscoveryStudio program
